# Numerical Investigation on Effect of Chamfering on Mechanical Behaviors in Continuous Network Composite

**DOI:** 10.3390/ma18204810

**Published:** 2025-10-21

**Authors:** Tao Li, Tianzi Wang, Jianchao Li, Cheng Liu, Bowen Gong, Wenting Ouyang, Likun Wang, Sainan Ma, Zhong Zheng, Bo Yuan, Huan Wang, Xiang Gao

**Affiliations:** 1 Ningbo Global Innovation Center, Zhejiang University, Ningbo 315100, China; 22460553@zju.edu.cn (T.L.); wanglk01@zju.edu.cn (L.W.); sainanma@zju.edu.cn (S.M.); 2 Institute for Composites Science Innovation (InCSI), School of Materials Science and Engineering, Zhejiang University, Hangzhou 310027, China; 12326064@zju.edu.cn (T.W.); lijianchao@zju.edu.cn (J.L.); 0624448@zju.edu.cn (C.L.); 11726046@zju.edu.cn (B.G.); 12126008@zju.edu.cn (W.O.); hwang2014@zju.edu.cn (H.W.); 3 School of Materials and Energy, Foshan University, Foshan 528000, China; zhengzhongmust@163.com; 4 Institute of Intelligent Manufacturing Technology, Shenzhen Polytechnic University, Shenzhen 518055, China; yuanbo@szpu.edu.cn

**Keywords:** metal-matrix composites (MMCs), Finite element analysis (FEA), network architecture, cell chamfering

## Abstract

The network architecture has demonstrated considerable potential for enhancing the strength–ductility synergy in metal matrix composites (MMCs). Intuitively, the intersections of network layers are expected to induce a stress concentration, leading to premature brittle fractures. Introducing chamfers to round the network cells may mitigate the local stress concentration and thereby improve elongation. Here, a numerical simulation framework was developed to investigate the effect of chamfering on the mechanical behavior of a three-dimensional (3D) continuous SiC_3D_/Al composite with a network architecture. A Voronoi tessellation algorithm was employed to generate the continuous network structural SiC phase. By inducing ductile and brittle damage criterions in the matrix and reinforcement elements, respectively, the mechanical behavior can be predicted via the finite element method (FEM). The predicted mechanical properties reveal an unexpected trend: chamfering results in a simultaneous reduction in both strength (from 367 MPa to 312 MPa) and elongation (from 4.1% to 2.0%). With chamfering, the enlarged intersection of the network layer bears a lower load, whereas the narrower network plates exhibit higher stress concentrations. As a result, the overall load-bearing capacity of the SiC_3D_ reinforcement decreases monotonically with an increasing chamfer size *f*. Furthermore, the non-uniform stress distribution promotes the premature fracture of the SiC_3D_, which reduces elongation. Additionally, the crack deflection behavior is suppressed in the chamfered models, leading to decreasing energy dissipation. This unanticipated outcome highlights an important architectural design principle: maintaining uniform geometric dimensions is critical for achieving optimal composite performance.

## 1. Introduction

Reticulated architectures are highly versatile and have been primarily employed in applications in fluid transport [1], heat transfer [2], damping [3], impact resistance [4], and sound absorption [5]. Incorporating such architectures into metal matrix composites (MMCs) also leads to excellent comprehensive mechanical properties. Inspired by this concept, lightweight co-continuous structures, i.e., closed-cell foams [6], open-cell foams [7], minimal surface scaffolds [8], and lattice trusses [9], have attracted widespread attention. These structures offer a promising approach to achieve weight reduction goals, due to their ideal enhancing effects in MMCs. Therefore, microstructural design is a key challenge in the development of advanced high-performance MMCs.

Recently, network architectures have demonstrated significant potential for enhancing the strength–ductility synergy in metal matrix composites (MMCs) [10]. The quasi-continuous distribution of reinforcements improves the load-bearing capacity and thus contributes to strengthening [11]. Yet, such reinforcements may also induce premature fractures. On the other hand, the monolithic matrix blunts micro-crack tips, thereby inhibiting crack propagation [10,11]. Therefore, network architecture design offers a feasible approach for simultaneously enhancing strength and elongation.

Unfortunately, architectural designs often lead to enhanced strength at the expense of reduced elongation. Yu et al. [12] fabricated a networked graphene oxide (GO)-reinforced TA1 composite via spark plasma sintering (SPS), which exhibited a 35% increase in strength but a 27% reduction in elongation compared to the TA1 matrix alloy. In composites such as CNT/Al [13], GNR/Al [13], and Al2O3/Al [14], the network architectures resulted in a significant strength improvement with only a slight decreasing elongation. These findings suggest that architectural optimization could further enhance both strength and elongation.

Huang et al. produced quasi-continuous TiBw/TC4 [15] and TiBw/Ti [10] network composites. In the TiBw/TC4 system, strength was improved while elongation was compromised [15]. However, through structural design, a simultaneous enhancement of strength and elongation was achieved in the TiBw/Ti system [10], indicating that the compatibility between the architectural design and composite system is crucial. Zhang et al. [16] developed a graphene/Cu composite with a network distribution, which showed significant improvements in both strength (↑32%) and elongation (↑93%) compared to its homogeneous counterpart. Network-structured Al_3_Ti/2024Al [17] and nano-ZrCp/2024Al [18] composites also exhibited notably enhanced strength and elongation compared to the 2024Al alloy. This further confirms that a well-matched composite system and architectural design are essential for simultaneously strengthening and toughening.

The reticulated Ti_2_AlC/TiAl composite demonstrated higher strength and elongation at both room and elevated temperatures than monolithic TiAl [19]. Mohammad [20] fabricated reticulated co-continuous NiAl-TiC composites, which exhibited higher flexural strength (*G_f_*), fracture toughness (KIC), and hardness compared to discrete particulate network composites. These results emphasize that controlling reinforcement clustering with very high local contents within the network architecture is key to achieving a high performance.

Detailed structural features also play an important role. In network composites, the monolithic matrix cells typically exhibit polyhedral shapes with sharp corners, leading to stress concentration and premature micro-cracking during deformation. Therefore, utilizing rounded matrix cells with blunted corners may improve elongation. The experimental investigation of these geometric effects remains challenging due to difficulties in controlling structural details. Thus, the finite element method (FEM) has become a promising tool, as evidenced by its extensive application in architectural composite research [11,21,22]. For conventional interpenetrating SiC/Al composites, geometry is often reconstructed from real materials, limiting flexibility in structural variations [23,24]. Hence, there is a growing need for digital modeling techniques that can generate customizable architectures efficiently using mathematical algorithms with few parameters. Soyarslan et al. [25,26] developed a controllable 3D stochastic interpenetrating structure generation method, while Ghazi et al. [27,28] proposed a tunable closed-cell modeling strategy. Such approaches enable the establishment of structure–property relationships [11,21,29], facilitating the design of high-performance composites.

In this work, a three-dimensional (3D) continuous SiC phase (SiC_3D_) with a network architecture was established with a tunable chamfer size to regulate the local stress concentration. So, microstructure-based composite models with various geometric parameters were built. The mechanical behaviors were predicted via the FEM. The mechanical behavior and performance were investigated by analyzing the stress/strain fields and stress–strain curves. An unanticipated effect of chamfering on intensifying non-uniform stress distributions was found, which is harmful to comprehensive mechanical properties.

## 2. Establishment of FEM Models

### 2.1. Geometrical Modeling Method

**Figure 1:**
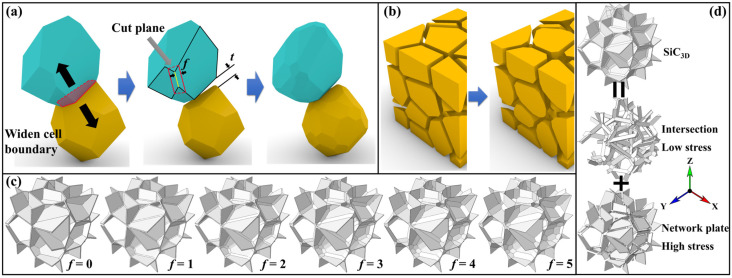
Rounded cell establishment process and the geometry models: (**a**) Chamfering process, (**b**) Comparison between origin and rounded cell model, (**c**) Various SiC_3D_ models with chamfer size *f* ranging from 0 to 5, (**d**) Schematic of intersection and plate parts of SiC_3D_.

In this work, the polyhedral shapes are rounded through a chamfering process (Figure 1a). Each polyhedral cell shrinks as all its surfaces move inward along their respective normal directions. During this process, the inter-cell distance (*t*) is used to widen the matrix cell boundary, i.e., controlling the cell contraction. The edges of the polyhedral cells were trimmed along planes defined by a cut plane (defined by red lines), as shown in Figure 1a. These red solid lines are parallel to the original edge (yellow line) and offset by a fixed distance, referred to the chamfer size (*f*). This procedure results in the generation of rounded polyhedral cells (Figure 1b). The geometry feature of reinforcement was also controlled by this process.

The dimensions of the model were set to 100 μm. A Voronoi tessellation algorithm was employed to generate the polyhedral cells. Our previous simulation results determined that a minimum of 20 cells is necessary to ensure statistical reliability [21]. Consequently, a count of 40 cells was selected for all models in this work to ensure the representativeness of the geometry models. In this work, the volume fraction of the SiC_3D_ phase is governed by two geometrical parameters. The chamfer size *f* was applied to control the geometry morphology of the models, while the SiC_3D_ volume fraction was held constant at 10% through the regulation of the inter-cell distance *t*. The increasing chamfering size *f* leads to a short inter-cell distance *t*. Thus, the lowest *t* was limited to no less than one order of magnitude of the model without chamfering. Therefore, the chamfer size *f* ranges from 0 to 5, so that the dynamically regulated inter-cell distance *t* decreases from 1.451 μm to 0.145 μm. The chamfered SiC_3D_ model can be divided into intersection and network plate parts (Figure 1d). A complete listing of the corresponding parameters and resultant geometric features is presented in Table 1. The geometry feature affects the load-bearing capacity of the reinforcement.

### 2.2. Material Properties

In this work, the composite 10 vol.% SiC_3D_/6061Al-T6 was applied as a model material system to investigate the general effect of chamfering on the mechanical behaviors in the composites with network architectures. The composition of 6061Al was Al 95.8–98.6 wt.%, Mg 0.8–1.2 wt.%, Si 0.4–0.8 wt.%, Cr 0.04–0.35 wt.%, and Cu 0.15–0.40 wt.%. The T6 aging process was employed by holding it at 170 °C for 10 h. The 6061Al-T6 matrix is a ductile material with a Young’s modulus of 69 GPa, a Poisson ratio of 0.33, a yield strength of 269 MPa, and a density of 2.7 g/cm^3^. The plasticity data was extracted from the experimental stress–strain curve (Figure S1).

Material failure is characterized by the complete loss of the load-bearing capacity due to the progressive degradation of the material stiffness. In the present numerical model, once damage initiation occurs, the mechanical response of an element is governed by stiffness degradation, described by the relation:

(1) $$\sigma =\left(1-D\right)\bar{\sigma }$$

where *D* is the stiffness degradation factor, *σ* represents the degraded stress tensor, and $\bar{\sigma }$ denotes the stress tensor in the undamaged state. Clearly, an element loses its load-carrying capacity when *D* reaches 1. The element is removed from the mesh once all its section points have lost their load-bearing capacity.

The failure behavior of the matrix is modeled using a phenomenological ductile damage criterion. Damage initiation is triggered when the equivalent plastic strain *ε_p_^f^* of the matrix reaches 0.11, i.e., a value determined from the experimental stress–strain curve of 6061Al-T6 (Figure S1), where the plastic strain at failure is approximately 0.11. The plastic strain increment from damage initiation to complete failure (*ε^d^*) is set to 0.001. A linear damage evolution law, based on effective plastic displacement, is adopted for the aluminum matrix. The damage variable evolves as

(2) $$D_{Al}=L_{e}{\dot{\varepsilon }}^{p}/u^{d}=L_{e}{\dot{\varepsilon }}^{p}/L_{e}\varepsilon^{d}={\dot{\varepsilon }}^{p}/\varepsilon^{d}$$

where *L_e_* is the characteristic element length, ${\dot{\varepsilon }}^{p}$ is the equivalent plastic strain rate after damage initiation, and *u^d^* is the plastic displacement corresponding to the failure strain *ε^d^*.

The SiC phase is a typical brittle material, with a Young’s modulus of 427 GPa, a Poisson ratio of 0.17, and a density of 3.2 g/cm^3^. The brittle fracture model is commonly applied to ceramics, concretes, and brittle alloys. In this study, a brittle failure criterion is incorporated into the composite model to represent the fracture behavior of the SiC particles. The brittle fracture is assumed to initiate when the equivalent stress exceeds 2 GPa. Crack initiation is governed by a single tensile macro-fracture (Mode I cracking), while the post-initiation shear behavior (Mode II cracking) is accounted for through a shear retention model that describes the degradation of the shear stiffness:

(3) $$D\left(e^{ck}\right)=\frac{\rho }{1-\rho }G,\;\rho \left(e^{ck}\right)={\left(1-\frac{e^{ck}}{e_{max}^{ck}}\right)}^{p}$$

where *e^ck^* is the crack opening strain, and *p* and *e^ck^_max_* are shear retention-related parameters. Zhang et al. [30] numerically extrapolated these parameters by fitting the tensile curve of the 7% SiC/Al composite, resulting in an *e^ck^_max_* = 0.2 and *p* = 2. The previous observations on the SiCp/Al fracture surfaces indicate that the matrix is retained on the SiC surfaces. This implies that the SiC/Al interface has a strong bonding. Therefore, perfect interfacial cohesion is assumed in this work.

### 2.3. Boundary Conditions

A finite element model was established in ABAQUS using 4-node tetrahedral (C3D4) elements. A mesh convergence study, informed by previous simulations of SiC/Al composites, confirmed that element sizes below 0.01–0.02% of the model size produce mesh-independent results [30,31]. Therefore, a uniform mesh size of 1–2 μm was implemented, generating approximately 850,000 elements. To enhance computational efficiency, a semi-automatic mass scaling method with a factor of 10,000 was applied.

The boundary conditions were applied to constrain the normal surface directions during deformation. This mimics the real tensile deformation behaviors to ensure the representativeness of the models. Two specific points were defined: a fixed reference point O at (0, 0, 0) and a loading reference point RF at (100, 100, 100). To constrain the displacements of boundary nodes, a linear multi-point constraint equation was applied to restrict mechanical degrees of freedom, expressed as

(4) $$A_{1}u_{i}^{P}+A_{2}u_{j}^{Q}+\cdot \cdot \cdot +A_{N}u_{k}^{R}=0$$

where *P* denotes a node set, *u_i_^P^* is the nodal displacement variable at a node in set *P*, *i* indicates the degree of freedom, and *A_N_* is the coefficient defining the relative motion among nodes. Using this formulation, nodes on the surface *x* = 0 were fixed in the *x*-direction (*u_x_* = 0) via the constraint:

(5) $$u_{x}^{surface\;x=0}-u_{x}^{O}=0$$

Similarly, the surfaces *y* = 0 and *z* = 0 were constrained to have *u_y_* = 0 and *u_z_* = 0, respectively. Nodes on the surface *x* = 100 were tied to the loading point RF in the *x*-direction using

(6) $$u_{x}^{surface\;x=100}-u_{x}^{RF}=0$$

Analogous constraints were applied to the surfaces *y* = 100 and *z* = 100 to enforce uniform displacement in the corresponding directions. A displacement-controlled tensile load was applied along the x-axis at a reference point (RP), with a maximum nominal strain of 8%. The external tensile strain rate was set to 2 × 10^−4^ s^−1^.

The mechanical behavior of the SiC_3D_/Al models was simulated using the commercial finite element software ABAQUS/Explicit (v6.13, Dassault Systèmes, Paris, France). The feasibility [32] and reliability [33] of this numerical approach on the SiC/6061-T6 composite system has been validated in our previous works.

## 3. Results and Discussion

### 3.1. Tensile Properties of Composites with Network Architecture

**Figure 2:**
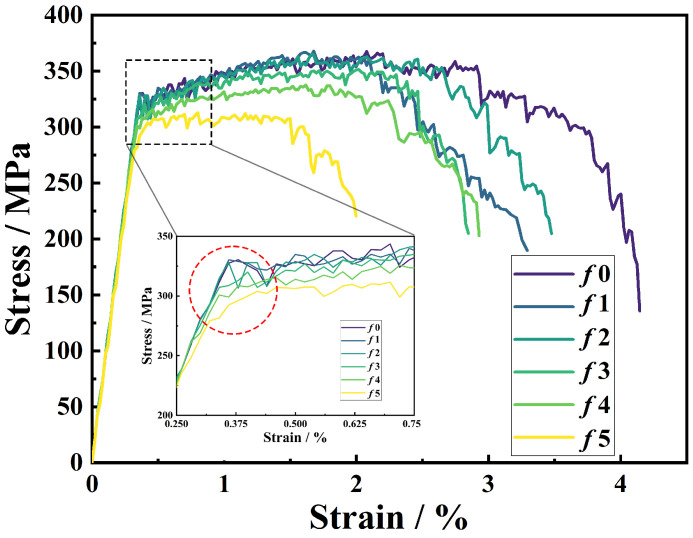
Comparison of predicted stress–strain curves of SiC_3D_/6061Al network composites with various chamfer sizes. The inset shows yield behaviors. The circle indicates different yield behaviors of the models.

Figure 2 presents the stress–strain curves of network composites with varying chamfer sizes *f*. The curves are truncated upon the formation of the main crack, as the subsequent behavior becomes less representative. In this work, the external nominal strain at main crack initiation (*ε^m^_xx_*) is applied to evaluate elongation.

In the network architecture, the monolithic matrix cells exhibit polyhedral shapes with sharp corners, which can induce local stress concentrations and lead to premature failure during deformation. To mitigate this, a chamfering process was introduced to round the matrix cells, thereby suppressing the localized stress concentration. As indicated by the circle in Figure 2, small chamfer sizes result in significant fluctuations in the stress response. This initially reveals that chamfering may improve elongation.

However, an unexpected trend is observed in the stress–strain curves: both strength (367 MPa → 312 MPa) and elongation (4.1% → 2.0%) decline as the chamfer size *f* increases. Contrary to expectations that chamfering would enhance elongation by alleviating the stress concentration, the results show an unexpected reduction. Moreover, the chamfered models present a larger SiC/Al interface area (Table 1), which should promote more effective load transfer from the matrix to the reinforcement and thus potentially increase strength. But the measured strength is unexpectedly lower.

The work hardening rate (*r*) was also calculated. The true stress and (*σ_true_*) true strain (*ε_true_*) data was extracted. Then, Hollomon’s law was applied to analyze the work hardening rate (*r*):

(7) $$\sigma_{true}=K\varepsilon_{true}^{n}$$

where *K* is the strength coefficient, and *n* is the strain hardening exponent. The *K* and *n* were obtained by fitting the *σ_true_*-*ε_true_* curves (*ε_xx_* ranging 0.5–1.5%). The related results are listed in Table S1. Then, the work hardening rate (*r*) can be calculated by

(8) $$r=K\cdot n\cdot \varepsilon_{true}^{n-1}$$

It is obvious that the work hardening rate (*r*) remains notably low with large chamfer sizes in the plastic stage (Figure S2).

These anomalous findings highlight the need for further investigation into the load-bearing capacity of the SiC_3D_ reinforcement and the damage evolution mechanisms in chamfered models to elucidate the unanticipated effect of the chamfering on the network architecture.

### 3.2. Load-Bearing Capacity of SiC_3D_

In the composite system, the tangent moduli of the matrix and reinforcement dominate the load-bearing behaviors of the two phases. The modulus of SiC is 427 GPa, which is much higher than that of Al (69 GPa). Hence, the reinforcement bears a much greater load than the matrix counterpart. Therefore, separate comparisons of the stress distribution of the matrix and reinforcement are needed.

**Figure 3:**
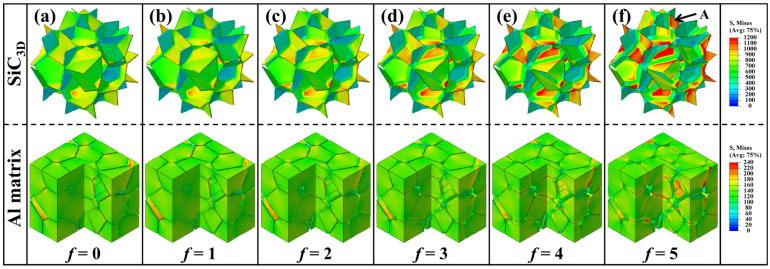
Load-bearing state of reinforcement in SiC_3D_/Al network composites with various chamfer sizes *f*. The arrow indicates high stress in network plate of SiC_3D_. The chamfer sizes are: (**a**) 0, (**b**) 1, (**c**) 2, (**d**) 3, (**e**) 4, (**f**) 5.

Figure 3 shows the von Mises equivalent stress distributions in the SiC_3D_ reinforcement and the Al matrix at an external strain of *ε_xx_* = 0.2% (within the elastic deformation stage). With various chamfer sizes *f*, the matrix exhibits a similar stress level, ranging from 100 to 160 MPa. This suggests that the strengthening effect originates primarily from the load-bearing capacity of the network-structural SiC_3D_.

As the chamfer size *f* increases, the stress distribution in the SiC_3D_ becomes increasingly non-uniform, with local values varying between 400 and 1200 MPa. The chamfered geometry models present the reduced width of the network plates (i.e., the inter-cell distance *t*, as shown in Table 1). This causes more of the load to be transferred to these plate regions (see Arrow A in Figure 3f). Conversely, the chamfered regions (i.e., the intersections) exhibit lower stress levels.

Three typical regions exhibiting high, medium, and low stress differences across the different models were selected from Figure 3, with each model further divided into intersection and plate segments (Figure S3). The load-bearing states in these regions were extracted and are presented in (Figure S4). At an external strain of *ε_xx_* = 0.2%, the stress levels in the SiC_3D_ phase vary among the typical regions, yet a consistent relationship between the average stress and chamfer size can be observed. The stress in the intersection regions decreases monotonically with the increasing chamfer size, whereas that in the network plate regions increases monotonically (Figure S4a–c). To quantify the non-uniformity in the load-bearing capacity between these two types of regions, the average stress ratio of the plate to the intersection (*K_t_*) is applied, which is given by

(9) Kt=σPlate_avgσIntersection_avg

where *σ_Plate_avg_* and *σ_Intersection_avg_* denote the average stresses in the plate and intersection regions, respectively. As illustrated in Figure S4d, *K_t_* increases with the chamfer size, and this increase becomes more pronounced at larger chamfer sizes.

Cross-sectional diagrams (Figure S5) reveal that thin plates are connected to V-shaped intersection bars, leading to a transfer of stress from the intersections to the plates. Geometrically, a larger chamfer size results in coarser intersections and thinner plates, which promotes stress transfer dramatically.

However, the load-bearing capacity also depends on the volume of the high-stress zone. Table 1 indicates that the volume of the intersection regions increases significantly with the chamfer size. Although the maximum local stress is highest in the model with *f* = 5, the overall load-bearing capacity of the SiC_3D_ does not necessarily improve. Moreover, the relatively uniform stress distribution in the SiC_3D_ (500–900 MPa) for the *f* = 0 model may lead to delayed brittle cracking, whereas the highly localized stress concentrations indicate a tendency for premature fractures in the *f* = 5 model. This stress localization may partly explain the reduced elongation observed at larger chamfer sizes. Nevertheless, the qualitative analysis of stress contours remains insufficient to explain the load-bearing behavior of the SiC_3D_ with a network architecture.

To evaluate the strengthening effect in composites with a continuous network architecture, the load-bearing capacity of the reinforcement phase was examined using a stress concentration factor. Based on the rule of mixtures, the effective stress of the composite is expressed as

(10) $$\sigma_{eff}=\left(\sigma_{SiC\_avg}V_{SiC}+\sigma_{Al\_avg}V_{Al}\right)/V_{Composite}$$

where *σ_eff_* is the effective stress of the composite, *V_SiC_* and *V_Al_* are the volume of the reinforcement and matrix, respectively, *σ_SiC_avg_* and *σ_Al_avg_* are the average stress of the reinforcement and matrix, respectively, and *V_Composite_* is the total volume of the composite, which is

(11) $$V_{Composite}=V_{SiC}+V_{Al}$$

**Figure 4:**
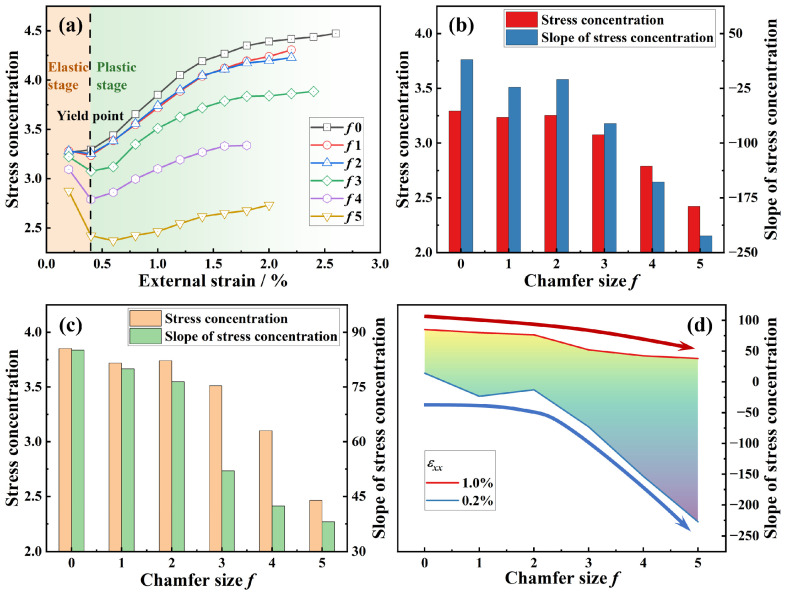
Stress concentration of SiC_3D_: (**a**) *R_SiC_*-*ε_xx_* evolution curves, (**b**) *R_SiC_* and the slope evolution response to chamfer size *f* at *ε_xx_* = 0.2%, (**c**) *R_SiC_* and the slope at *ε_xx_* = 1.0%, and (**d**) *R_SiC_*-*f* evolution at elastic (*ε_xx_* = 0.2%) and plastic (*ε_xx_* = 1.0%) stage.

The average stress of the matrix and reinforcement are defined as

(12) $$\sigma_{Al\_avg}=\frac{\sum_{i=Al}V_{i}\sigma_{i}}{\sum_{i=Al}V_{i}},\;\sigma_{SiC\_avg}=\frac{\sum_{i=SiC}V_{i}\sigma_{i}}{\sum_{i=SiC}V_{i}}$$

where *V_i_* and *σ_i_* represent the volume and stress of each element in the respective phase. Following the approach of Roy et al. [34], the stress concentration factors for the matrix and reinforcement are defined as follows:

(13) $$R_{Al}=\frac{\sigma_{Al\_avg}}{\sigma_{eff}},\;R_{SiC}=\frac{\sigma_{SiC\_avg}}{\sigma_{eff}}$$

where *R_Al_* and *R_SiC_* quantify the inhomogeneous stress distribution in the matrix and reinforcement, respectively. The evolution of *R_SiC_* with applied external strain is shown in Figure 4a. A strong influence of the chamfer size *f* on the *R_SiC_*–*f* relationship is evident.

In the elastic stage, both the matrix and reinforcement deform elastically, so *R_SiC_* is expected to remain constant. With the absence of chamfering, the stress distribution within SiC_3D_ is relatively uniform, without a significant stress concentration. However, the stress contour in Figure 3a reveals that the matrix adjacent to the SiC/Al interface experience higher stress levels compared to the interior counterpart. This indicates localized plastic deformation in this matrix region. As a result, the tangent modulus decreases markedly, leading to increased load transfer to the SiC_3D_ reinforcement and a corresponding rise in *R_SiC_* with increasing external strain, even in the nominal elastic stage.

With increasing chamfer size *f*, the stress distribution in SiC_3D_ becomes increasingly non-uniform (Figure 3). The plate regions of the network bear significantly higher loads, while their volume decreases considerably (Table 1). These highly stressed plate regions reach their fracture strength at an early stage, resulting in premature brittle failure. This reduces the overall load-bearing capacity of SiC_3D_ during elastic deformation. So, a declined *R_SiC_*-*ε_xx_* trend was shown in the elastic stage (Figure 4a). Although the plate regions sustain high stresses, their diminished volume due to chamfering leads to a simultaneous decrease in both *R_SiC_* and its slope of increase with external strain (Δ*R_SiC_*/Δ*ε_xx_*), as shown in Figure 4b.

In the plastic stage, *R_SiC_* increases with applied external strain *ε_xx_* (Figure 4a), owing to the sharp decline in the tangent modulus of the Al matrix. However, larger chamfer sizes exacerbate the non-uniformity of the stress in SiC_3D_, promoting more severe brittle fractures and considerably reducing the load-bearing efficiency of the reinforcement. As illustrated by the declining trend of the *R_SiC_*–*f* evolution (Figure 4c), both *R_SiC_* and Δ*R_SiC_*/Δ*ε_xx_* decrease with increasing *f*. Figure 4d visually summarizes the detrimental effect of chamfering on Δ*R_SiC_*/Δ*ε_xx_*. Since *R_SiC_* reflects the strengthening contribution of SiC_3D_, the reduction in Δ*R_SiC_*/Δ*ε_xx_* with *f* reflects the decreased work hardening rate (*n*) observed in the stress–strain curves (Figure 2).

### 3.3. Damage Behavior of SiC_3D_/Al with Network Architecture

**Figure 5:**
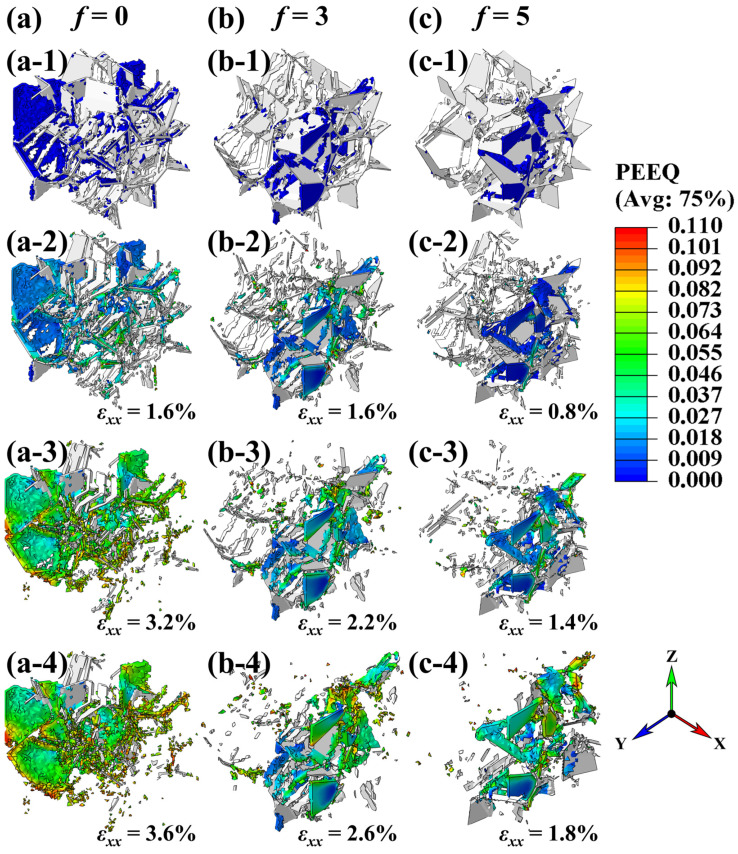
The equivalent plastic strain contours of models with (**a**) *f* = 0, (**b**) *f* = 3, and (**c**) *f* = 5. (**a-1**–**c-1**) depict the damaged elements that shape the main crack at *ε_xx_* = 0. (**a-2**–**c-2**) present the premature fracture of SiC_3D_ in the plastic stage. The insets (**a-3**–**c-3**), (**a-4**–**c-4**) show the dramatically damage behavior of the matrix that the main crack initiates.

Figure 5 displays the equivalent plastic strain distributions in the network-structural composites with various chamfer sizes. Insets (a-1) to (c-1) show the damaged elements of the models, illustrating the main crack morphologies at *ε_xx_* = 0. The blue elements represent the matrix, while the white regions correspond to the reinforcement. It is noteworthy that matrices with larger chamfer sizes (higher *f*) exhibit reduced damage, implying that the brittle fracture of the SiC_3D_ reinforcement dominates the crack propagation. This behavior can be attributed to the localized stress concentration within the plate-like regions of the SiC_3D_ network, which facilitates premature brittle failure.

The orientation of the network layers relative to the loading direction plays a critical role in determining the load-bearing capacity of each network plate. A comparison between insets (a-2) and (a-1) in Figure 5 reveals that plates aligned parallel to the loading direction experience higher stress levels and are more prone to premature failure. In contrast, for the model with a chamfer size of *f* = 5, the highly non-uniform stress distribution within SiC_3D_ leads to exceptionally high stress in the plate regions (as seen in Figure 3f), resulting in a severe brittle fracture at *ε_xx_* = 0.8% (Figure 5c-2). The model without chamfering (i.e., *f* = 0) exhibits significantly fewer fractured SiC elements (Figure 5a-2), owing to its more uniform and lower stress distribution. The stress contours indicate that primary cracks initiate at fractured SiC regions and are subsequently blunted by the adjacent ductile matrix cells. This crack-blunting mechanism effectively delays the propagation of the main crack, thereby enhancing elongation.

As shown in insets (a-3) to (c-4) in Figure 5, the main crack initiation coincides with the deletion of a large number of matrix elements. The corresponding nominal external strain at crack initiation (*ε_xx_*) decreases with the increasing chamfer size *f*, which is consistent with the reduced elongation observed in the stress–strain responses (Figure 2). Moreover, Figure 5a-4 demonstrates that the matrix in the *f* = 0 model undergoes extensive and relatively uniform plastic deformation, reflected in a higher and more sufficient plastic strain. This implies a lower stress concentration at the main crack tip. In contrast, the main crack paths in Figure 5b-4,c-4 are relatively straight. The pronounced crack deflection observed in the unchamfered model enhances energy dissipation through a zigzag crack path, which is beneficial for improving elongation.

In our previous experimental study on SiCp/Al network composites, it was observed that a slight increase in strength (from 365 MPa to 395 MPa) was accompanied by a significant reduction in elongation (from 4.5% to 2.9%) compared to the homogeneous counterpart [35]. This can be attributed to the non-uniform network width, particularly the wider intersections. This indicates that the architectural design still requires optimization. A similar phenomenon is observed in the chamfered SiC_3D_ models in the present work. In addition, Prasad et al. [36] fabricated a SiC/Al composite with a round network architecture. Unfortunately, this round geometry feature led to a simultaneous decrease in strength and elongation. So, the previous experimental results also supported the idea that a large chamfer size decreases the elongation.

Notably, successful examples of architectural designs in metal matrix composites [10,16,17,18] consistently employ a uniform network width. In those cases, both strength and elongation were improved simultaneously compared to the unreinforced matrix alloy. This reveals that maintaining uniform structural dimensions is a critical factor for achieving superior mechanical properties in composites with network architectures.

However, the number of successful cases is still limited. The actuality is that architecture design is not a panacea. More experimental results demonstrated that the strengthening effect of the architecture design sacrificed elongation [15,35]. Moreover, Lin et al. [37] and Zhang et al. [16], coming from the same group, prepared network architectures in CNT/Al and GNP/Cu composites, respectively, with the same structural design and manufacturing strategies. The CNT/Al network composite presents a higher strength with reduced elongation compared to that of the Al matrix. However, the GNP/Cu showed simultaneously enhanced strength and elongation. Huang et al. induced network architecture designs in the TiBw/Ti [10] and TiBw/TC4 [15] composite systems. Comparing these to the homogeneous ones, the TiBw/Ti network showed higher strength and elongation, but the architecture enhanced the strength of TiBw/TC4 while sacrificing elongation. These works show that the architecture design must be complemented by a well-matched composite system. So, this is still an open question, requiring lots of further systematic investigations.

Owing to the challenges associated with fabricating closed-cell foam structures in the SiC/Al system, the experimental validation of the present simulation remains limited. Nevertheless, this work establishes a viable approach for predicting the mechanical effects of chamfered network architectures. It thus provides a useful tool for future studies on architectural design in the given composite system.

## 4. Conclusions

In summary, while it is intuitively expected that intersections in the network architecture would cause stress concentrations and lead to premature brittle fractures, thereby reducing elongation, it is expected that chamfering might alleviate this issue. However, an unanticipated phenomenon is observed: chamfering results in a simultaneous decrease in both strength (from 367 MPa to 312 MPa) and elongation (from 4.1% to 2.0%). This is attributed to the increased size of the intersection regions relative to the plate segments, which promotes non-uniform stress distribution. As a consequence, the overall load-bearing capacity of the SiC_3D_ reinforcement decreases monotonically with the increasing chamfer size *f*. Furthermore, the inhomogeneous stress state accelerates the premature fracture in SiC_3D_, leading to a further reduction in elongation. The main cracks initiate earlier and propagate along straighter paths, thereby dissipating less energy. These findings emphasize the importance of maintaining uniform architectural dimensions in network composites as a key optimization strategy for achieving well-balanced mechanical properties.

## Figures and Tables

**Table 1:** The geometrical parameters and structural features.

Parameters/Features	Values					
Chamfer size (*f*)/μm	0.00	1.00	2.00	3.00	4.00	5.00
Inter-cell distance (*t*)/μm	1.451	1.390	1.217	0.944	0.583	0.145
Foam volume/μm^3^	100,000	99,976	99,976	99,978	99,964	99,970
Network plate volume/μm^3^	100,000	71,952	54,439	36,714	19,824	4331
Intersection volume/μm^3^	0	28,024	45,537	63,264	9014	95,639
Interface area/μm^2^	133,369	130,719	128,579	126,928	125,542	124,628

## Data Availability

The original contributions presented in this study are included in the article/supplementary material. Further inquiries can be directed to the corresponding author.

## Supplementary Materials

The following supporting information can be downloaded at https://www.mdpi.com/article/10.3390/ma18204810/s1, Table S1: The strength coefficient *K* and strain hardening exponent *n* of the models (*ε_xx_* 0.5–1.5%); Figure S1: Experimental stress–strain curve of 6061Al-T6; Figure S2: Work hardening rate of the models with various chamfer size; Figure S3: Three typical regions to comparing load-bearing state between intersection and plate parts. Arrow A is high stress region, B indicates medium stress region, C shows low stress region; Figure S4: Stress level in SiC_3D_ of three typical regions. (a) High-stress region, (b) Medium-stress region, (c) Low-stress region, (d) Stress ratio of plate part to intersection counterpart; Figure S5: Morphology diagram of intersection and plate parts in SiC_3D_.
