# Supplementary material for: Numerical Investigation on Effect of Chamfering on Mechanical Behaviors in Continuous Network Composite
Source: Materials (Basel). 2025 Oct 21;18(20):4810. doi: 10.3390/ma18204810 (PMC12565935; doi:10.3390/ma18204810)
Supplement: Supplementary file 1 [file materials-18-04810-s001.zip › materials-3908503-supplementary.pdf]

materials

Supplementary Materials

Numerical Investigation on Effect of Chamfering on Mechanical Behaviors in Continuous Network Composite

Tao Li [1,2], Tianzi Wang [2], Jianchao Li [2], Cheng Liu [2], Bowen Gong [2], Wenting Ouyang [2], Likun Wang [1], Sainan Ma [1], Zhong Zheng [3], Bo Yuan [4], Huan Wang [2] and Xiang Gao [1,*]

[1] Ningbo Global Innovation Center, Zhejiang University, Ningbo 315100, China; 22460553@zju.edu.cn (T.L.); wanglk01@zju.edu.cn (L.W.); sainanma@zju.edu.cn (S.M.)
[2] Institute for Composites Science Innovation (InCSI), School of Materials Science and Engineering, Zhejiang University, Hangzhou 310027, China; 12326064@zju.edu.cn (T.W.); lijianchao@zju.edu.cn (J.L.); 0624448@zju.edu.cn (C.L.); 11726046@zju.edu.cn (B.G.); 12126008@zju.edu.cn (W.O.); hwang2014@zju.edu.cn (H.W.)
[3] School of Materials and Energy, Foshan University, Foshan 528000, China; zhengzhongmust@163.com
[4] Institute of Intelligent Manufacturing Technology, Shenzhen Polytechnic University, Shenzhen 518055, China; yuanbo@szpu.edu.cn
* Correspondence: gaoxiang1986@zju.edu.cn

 https://doi.org/10.3390/ma18204810

Table S1. The strength coefficient *K* and strain hardening exponent *n* of the models (ε_{xx} 0.5%-1.5%).

Chamfer size (*f*) / µm	*K*	*n*
0.0	5357832.141	0.09256
1.0	5850455.035	0.11026
2.0	5380543.773	0.0937
3.0	4944567.218	0.0793
4.0	4534022.171	0.06814
5.0	3302432.909	0.01337

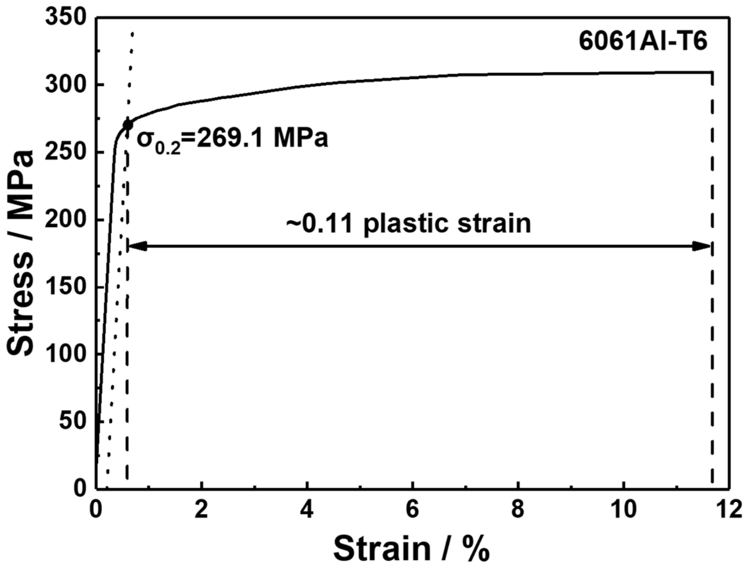

Figure S1. Experimental stress–strain curve of 6061Al-T6.

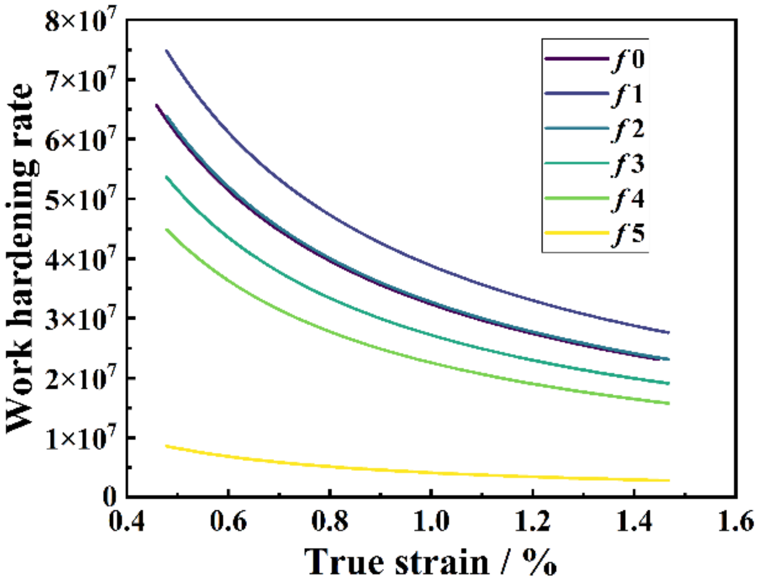

Figure S2. Work hardening rate of the models with various chamfer sizes.

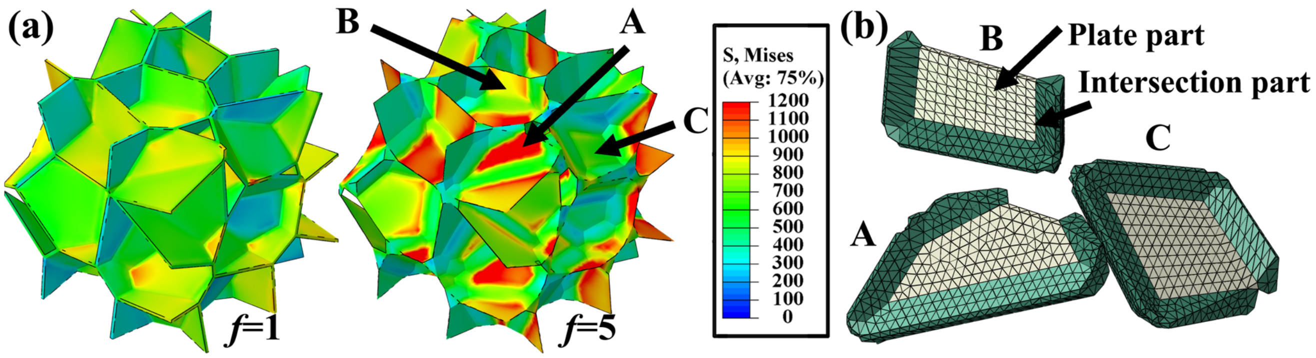

Figure S3. Three typical regions comparing load-bearing state between intersection and plate parts: (**a**) Load-bearing state of SiC_{3D} in models with chamfer size 1 and 5, (**b**) The geometries of regions A, B and C. Arrow A is high-stress region, B indicates medium-stress region, and C shows low-stress region.

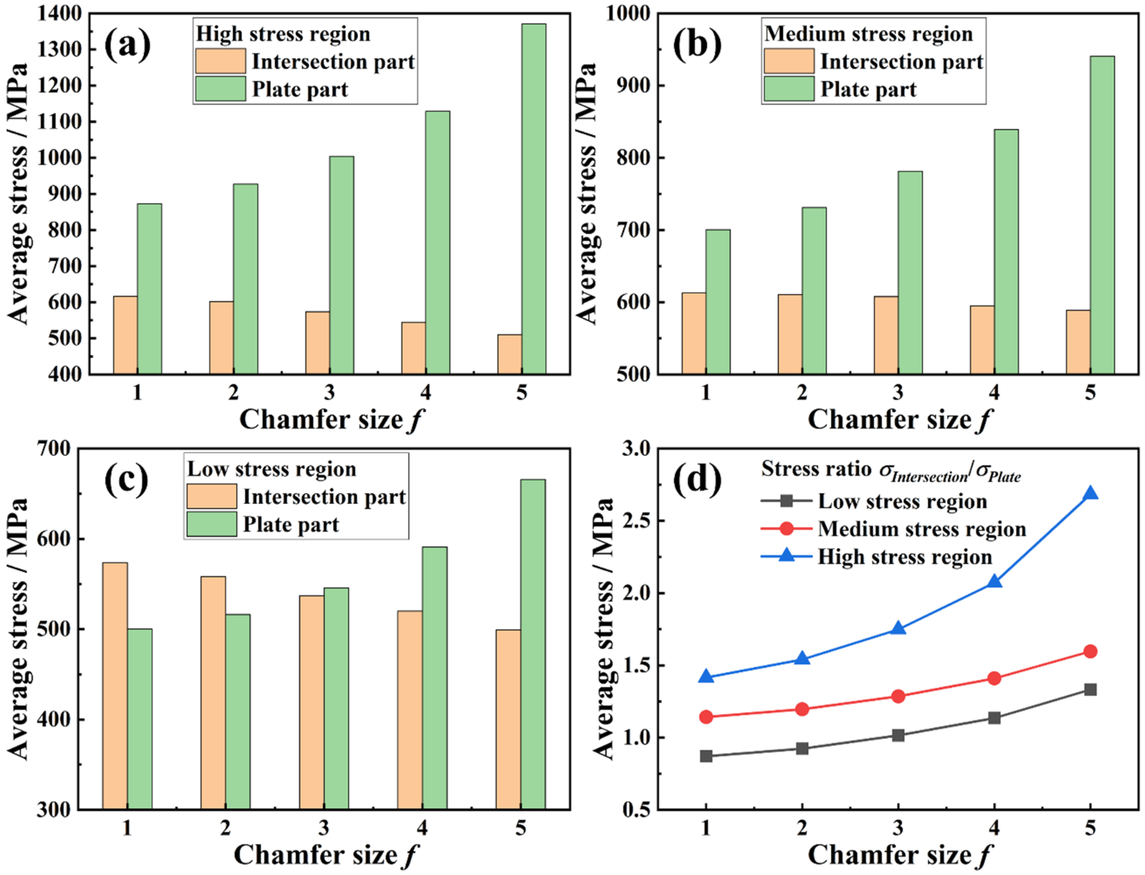

Figure S4. Stress level in SiC_{3D} of three typical regions. (**a**) High-stress region, (**b**) medium-stress region, (**c**) low-stress region, and (**d**) stress ratio of plate part to intersection counterpart.

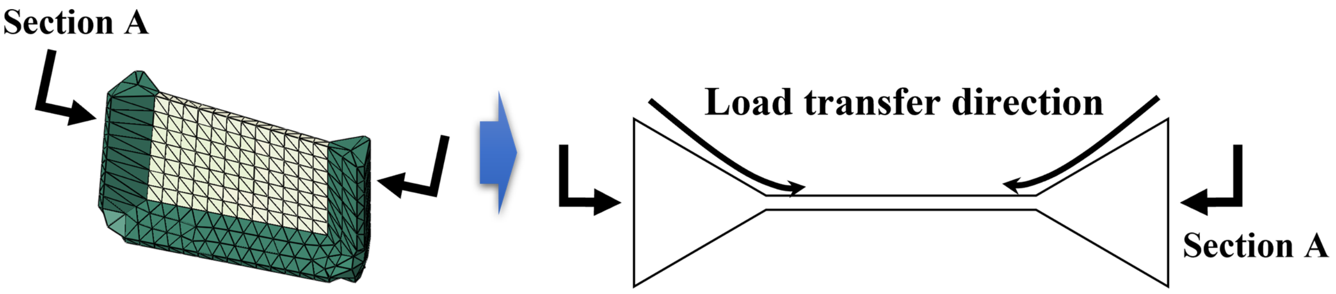

Figure S5. Morphology diagram of intersection and plate parts in SiC_{3D} leading to load transfer from intersection to plate part.
